# Assessing the impact of the COVID-19 pandemic on the mental health of female entertainment workers in Cambodia: a group model building approach

**DOI:** 10.3389/fpubh.2024.1336785

**Published:** 2024-11-21

**Authors:** Calida S. Chua, John P. Ansah, Sovanvorleak Tep, Sreymom Oy, Mengieng Ung, Siyan Yi

**Affiliations:** ^1^Saw Swee Hock School of Public Health, National University of Singapore and National University Health System, Singapore, Singapore; ^2^Center for Community Health Integration, Case Western Reserve University, Cleveland, OH, United States; ^3^Graduate School of Health Innovation, Kanagawa University of Human Services, Tonomachi, Kawasaki, Japan; ^4^Public Health Program, College of Education and Health Sciences, Touro University California, Vallejo, CA, United States

**Keywords:** COVID-19, mental health, female sex worker, group model building, low-and middle-income country, vulnerable population

## Abstract

**Background:**

The COVID-19 pandemic has intensified global mental health challenges, particularly for vulnerable groups like female entertainment workers (FEWs), a critical HIV-affected population in Cambodia. Already facing pre-existing inequalities, FEWs encountered heightened difficulties due to the pandemic’s disruption of their livelihoods. Their susceptibility to mental health problems is exacerbated by poverty and the occupational stressors they regularly endure. This study aims to identify COVID-19-related factors adversely impacting the mental well-being of FEWs in Cambodia and explore potential interventions to mitigate these effects.

**Methods:**

In December 2021, we conducted a two-day workshop in Phnom Penh using a group model-building approach with stakeholders from diverse backgrounds to gather collective insights. We employed the qualitative system dynamics method of causal loop diagram mapping to visualize the factors affecting FEWs’ mental health. Based on participants’ perceptions and experiences, we constructed a causal loop diagram to develop a comprehensive systems perspective.

**Results:**

The workshop involved 27 stakeholders, including representatives from national institutions (*n* = 3), local NGOs (*n* = 17), an international NGO (*n* = 1), and the FEW community (*n* = 6). Our analysis identified 13 feedback loops highlighting key factors influencing the mental well-being of FEWs during the pandemic. These factors included the loss of family members, financial instability, fear of COVID-19 infection, the pressure of children’s homeschooling due to school closures, and food insecurity. In response, stakeholders proposed a range of interventions, including alternative vocational training, increased awareness of mental health issues, access to mental health services, and programs targeting gender-based violence within FEW communities.

**Conclusion:**

A collaborative, multi-sectoral approach is crucial to addressing the mental health challenges of FEWs in Cambodia. Prioritizing the establishment of accessible, affordable, and high-quality mental health services, alongside the implementation of targeted interventions, is essential to mitigate the negative mental health impacts of the pandemic and enhance the overall well-being of FEWs.

## Introduction

The COVID-19 outbreak severely impacted the world across physical, economic, psychological, and social dimensions. Declared a global pandemic by the World Health Organization (WHO) in March 2020, control measures to curb transmission have profoundly affected economies, communities, and individuals. COVID-19 has been called a “pandemic of inequality,” disproportionately affecting the poor and vulnerable (1). Vulnerable populations, already facing economic, social, and health disparities, are at greater risk of poor health outcomes, particularly during crises (2). The interplay of social determinants and vulnerability increases the likelihood of developing mental health conditions (3), which in turn can perpetuate social exclusion and disadvantage. Poor mental health can, in turn, further beget social exclusion and disadvantage. Public safety measures implemented during emergencies often exacerbate the existing social and health inequalities these populations face. Many of these interventions led to economic hardship and lifestyle disruptions, fostering feelings of helplessness, uncertainty, and grief (3). Consequently, there has been growing attention to the impact of both the disease and the response policies on individuals’ mental health.

In March 2022, the WHO reported a 25% increase in the global prevalence of depression and anxiety during the first year of the COVID-19 pandemic (4). Significant disruptions to service provision compounded this mental health burden, as healthcare resources were diverted to the pandemic response (5). Studies have shown that women are more likely to develop symptoms of mental disorders than men (4). However, data from lower- and middle-income countries (LMICs) and specific vulnerable groups remain limited.

Vulnerable populations often face significant structural barriers in accessing healthcare services, including vaccination, mental health care, and economic opportunities. Female entertainment workers (FEWs), a critical HIV-affected population in Cambodia, exemplify such vulnerabilities. The term “FEWs” emerged following the 2007–2008 global financial crisis, which led to widespread unemployment in Cambodia’s garment industry and drove many young women into the entertainment and informal sex sectors. This shift was further influenced by the 2008 ‘Law on Suppression of Human Trafficking and Sexual Exploitation,’ which banned brothel-based sex work (6). FEWs typically include young women employed at beer gardens, restaurants, karaoke bars, or massage parlors, as well as freelance sex workers who operate in public spaces, such as parks or streets, or on-call (7, 8). Our previous study found that 60.7% of FEWs engaged in transactional sex (8).

In 2019, the number of FEWs in Cambodia was estimated at around 70,000 (9). Most FEWs come from low-income rural families with limited education, including low health literacy (10), and many provide regular financial support for their families (11). They often face social stigma within their communities and have less social support compared to other women (12, 13). Studies have shown that FEWs experience similar stigmatization and criminalization as female sex workers (FSWs), regardless of whether they engage in transactional sex (14). FEWs, particularly those involved in sex work, are at higher risk for health issues, including mental health problems, due to occupational exposures such as unsafe sex practices, verbal abuse, forced alcohol consumption, and physical violence (15–17). Despite these known risk factors, mental health issues remain understudied in this population (18). Based on current evidence, data on the mental health of FSWs are likely the most relevant for understanding FEWs’ mental health, with studies reporting up to 75% of FSWs experiencing mental health disorders (19–21). Moreover, social determinants such as low income, gender, limited education, inadequate housing, and unemployment, along with structural factors like gender inequality, harassment, arrests by law enforcement (22), lack of community support, unsafe work environments, and poor access to health and social services, further exacerbate their mental health vulnerabilities (23).

In 2015, a study reported that 43.2% of FEWs in Cambodia experienced high levels of psychological distress, 19.5% reported suicidal thoughts, and 7.3% had attempted suicide in the past 3 months due to past experiences and current working conditions (10). Those reporting low autonomy in their work environments—such as being forced to drink or having clients request unprotected sex—experienced higher levels of distress (10). The evidence indicates that FEWs, particularly those involved in sex work, face worse mental health outcomes than the general population, with a higher prevalence of suicidal behaviors, anxiety, psychological distress, depression, and post-traumatic stress disorders (24–26). Additionally, studies suggest that the poor mental health of FSWs can lead to risky behaviors, including unsafe sex practices and substance abuse (10). A 2020 systematic review and meta-analysis found that FSWs in LMICs had a high prevalence of mental health problems linked to behavioral and social factors they commonly experienced (22). Key risk factors for suicidal behavior and mental disorders—such as inadequate housing, HIV and other sexually transmitted infections (STIs), gender-based violence (GBV), drug and alcohol use, low education, financial stress, and discrimination and stigma (16, 17, 27, 28)—were reported at elevated levels among FSWs. Inconsistent condom use, drug use, HIV and other STIs, and GBV were also strongly associated with poor mental health in this vulnerable population (22).

The Cambodian government implemented stringent measures in early 2020 to curb the spread of COVID-19 (29, 30). These measures had far-reaching consequences, particularly for sectors like entertainment and informal sex work, severely disrupting the lives and livelihoods of many FEWs. Even before the pandemic, FEWs faced multiple challenges, including economic vulnerabilities and occupational stressors. The COVID-19 crisis exacerbated these pre-existing difficulties. Lockdowns, restrictions, and reduced economic activity disrupted their work, causing financial strain and deepening their vulnerability to various hardships. The cumulative effect of these challenges had profound implications for the mental well-being of FEWs, as the heightened economic and social stressors during the pandemic likely contributed to increased psychological distress within this group.

The COVID-19 pandemic has exacerbated existing psychological issues globally, particularly among socially disadvantaged groups. A recent meta-analysis on the pandemic’s impact on mental health found that unemployment, being under 40 years old, and female gender were associated with increased psychological distress (31). The analysis also highlighted that the pandemic uniquely intensified health risks and stressors for women (32). In addition to the external stressors created by the pandemic—such as economic instability and health risks—concerns about food security, housing, and fear of contracting COVID-19 while working without social protection are likely to worsen existing mental health conditions among FEWs (23). Moreover, the additional societal roles typically associated with women, such as childcare and education, may become more burdensome during the pandemic, further contributing to mental distress.

Cambodia’s mental health services are already limited, even for the general population (33). Given the prevalent societal disparities in the country, FEWs are likely to face even more significant challenges in accessing mental health support. Therefore, this study aims to comprehensively identify the COVID-19-related factors adversely affecting the mental well-being of FEWs in Cambodia. It also seeks to pinpoint specific areas where targeted interventions can improve the mental health of this vulnerable group, addressing a critical healthcare concern within the context of the country’s constrained mental health infrastructure.

## Methods

### Group model building approach

Data for this analysis were collected as part of a needs assessment during the COVID-19 pandemic within an ongoing intervention study to improve access to GBV services. We employed the GMB approach, a system dynamics model-building process. GMB is a participatory approach that engages a diverse group of stakeholders to develop a deeper understanding of complex issues by integrating and assimilating their mental models into a holistic system description (34). It emphasizes the complexity and dynamics of interactions between system components and provides a framework for interdisciplinary and transdisciplinary approaches to address complex problems (35). Additionally, GMB introduces social dynamics, which can influence model quality, stakeholder engagement, and the likelihood that recommendations will be accepted and implemented (36).

This study also utilized the qualitative system dynamics method of causal loop diagram (CLD) mapping to visualize the impact of the COVID-19 pandemic on the mental health of FEWs. A CLD is a visual tool that illustrates the interrelationships between variables within a system, consisting of variables or concepts, links (or arrows), and their polarities. The CLD captures the complex interrelationships identified through the GMB process to pinpoint the feedback mechanisms driving observed behaviors. Variables represent factors or concepts describing the system, while links (or arrows) indicate the direction of causal relationships, with plus (+) and minus (−) signs denoting the polarity of these relationships. Table 1 provides a detailed description of the notations used in the CLD.

**Table 1:** Overview of notations for the causal loop diagram.

Symbol	Definitions and function in causal loop diagram
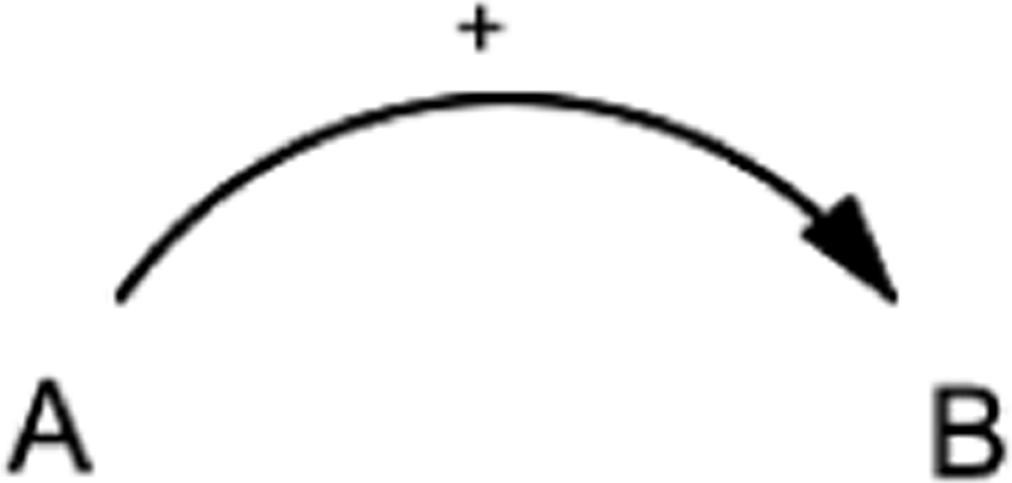	“+” means an increase (or decrease) in variable A leads to an increase (or decrease) in variable B (i.e., a change in the same direction).
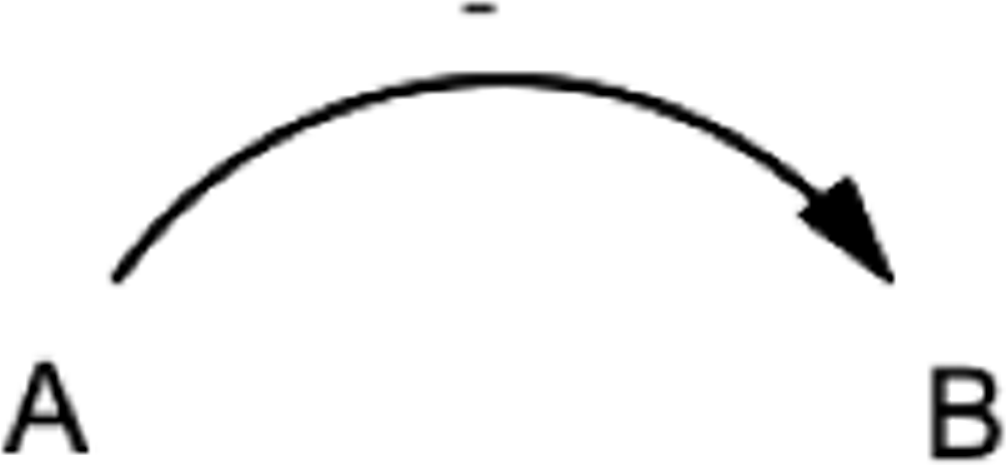	“-” means an increase (or decrease) in variable A leads to a decrease (or increase) in variable B (i.e., a change in the opposite direction).
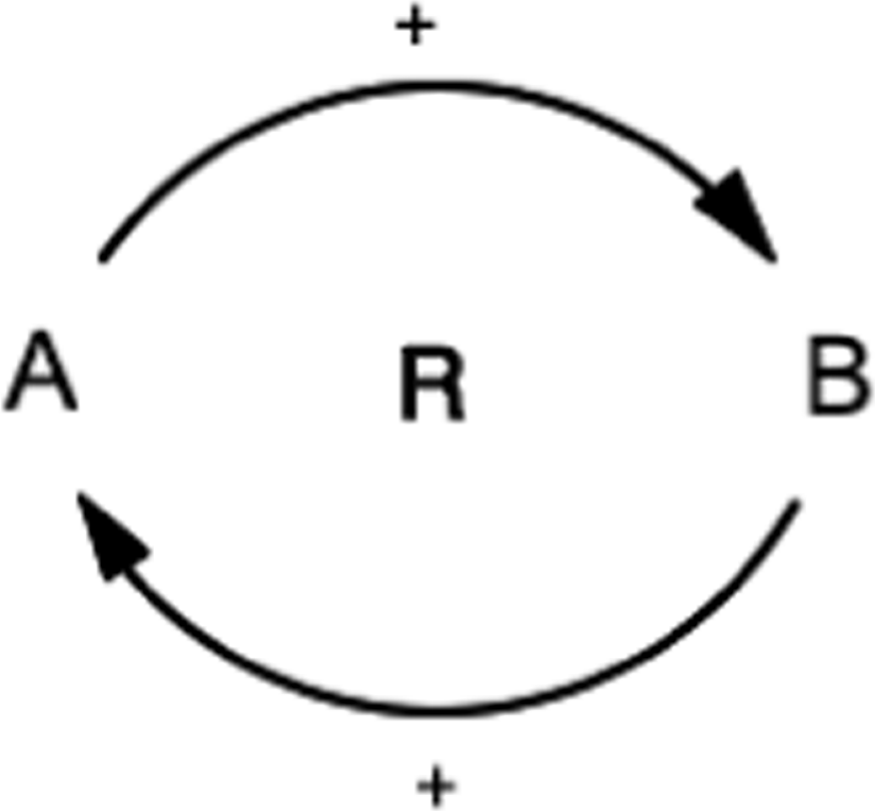	“R” indicates a reinforcing feedback loop whereby an increase (or decrease) in “A” causes an increase (or decrease) in “B,” which leads to a further increase (or decrease) in “A”. A reinforcing feedback loop results in subsequent exponential change (either growth or decay).
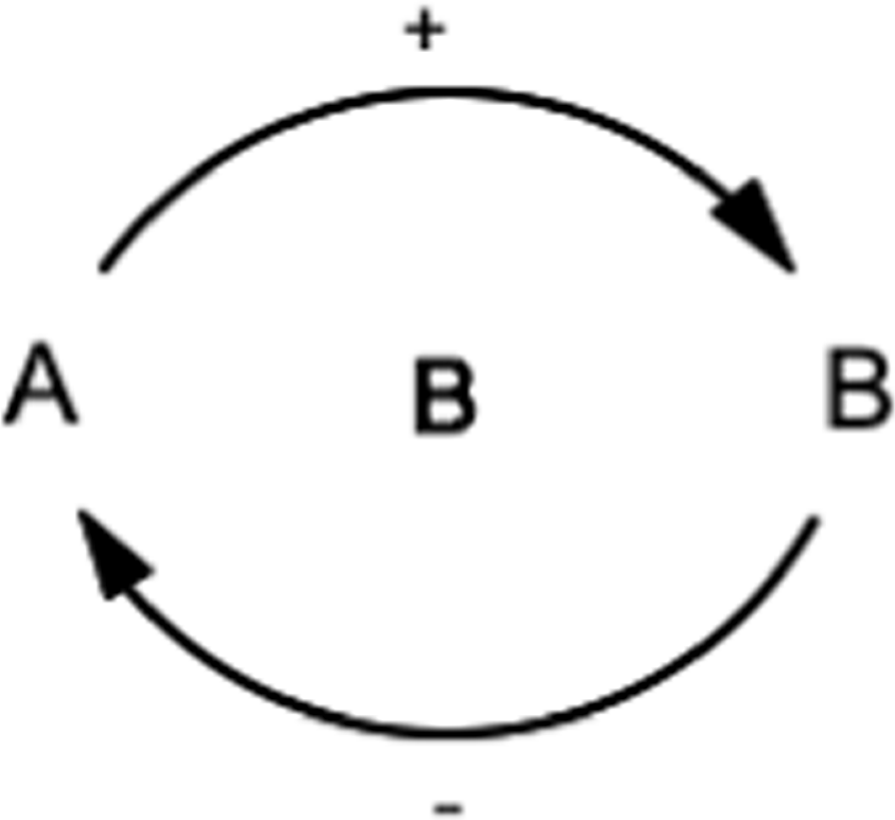	“B” indicates a balancing feedback loop whereby an increase (or decrease) in “A” causes an increase (or decrease) in “B,” which leads then to a decrease (or increase) in “A.” A balancing feedback loop results in changes toward equilibrium or moves the current state toward a desired state, seeking to stabilize a system.
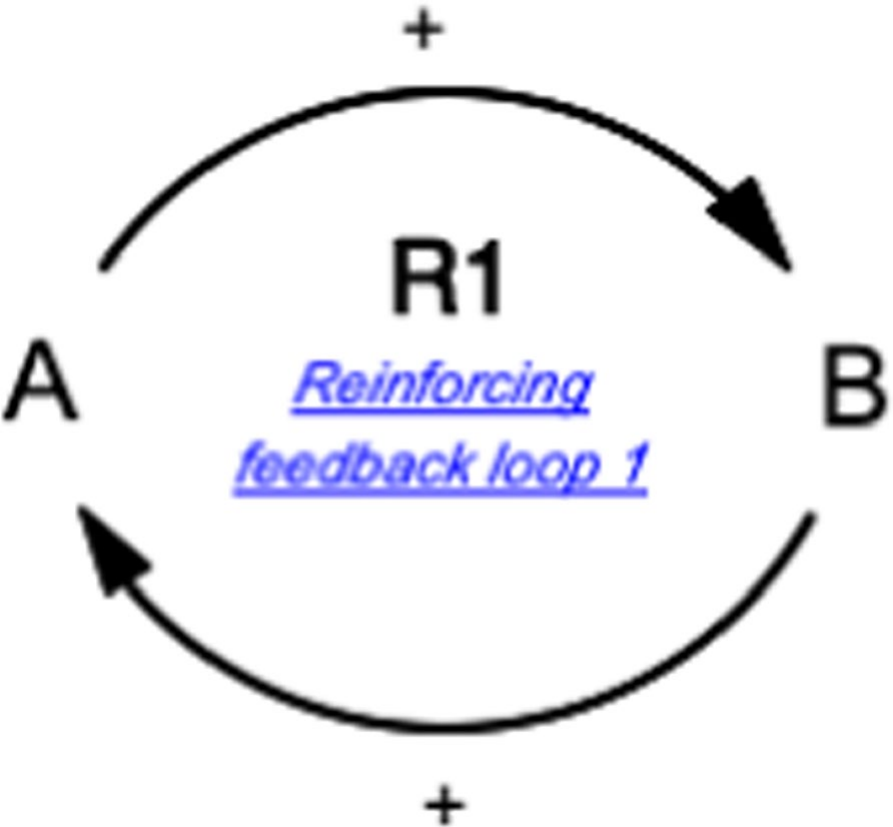	“R1” indicates reinforcing feedback loop number 1, and the feedback loop’s name describes it. All reinforcing feedback loops are numbered for easy identification. Supplementary Table S2 helps trace each loop’s causal pathway by following the numbering variables in each loop.
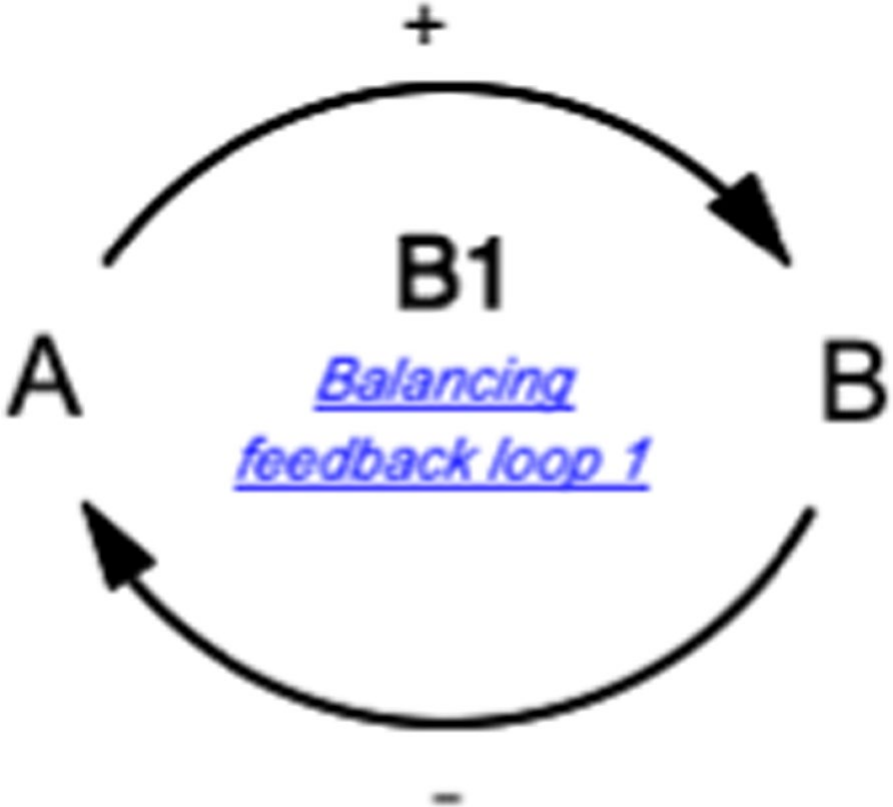	“B1” indicates balancing feedback loop number 1, and the feedback loop’s name describes it. All balancing feedback loops are numbered for easy identification. Table B in the appendix helps trace each loop’s causal pathway by following the numbering of variables in each loop.

Multiple stakeholders were engaged to incorporate diverse perspectives into a single conceptual model, capturing their understanding to achieve a comprehensive view of the relationship between the pandemic and mental health. The conceptual models developed with stakeholders were used to represent the dynamic relationships between outcomes of interest and underlying factors, providing a thorough insight into the complexity of the issue (37). This resultant model can enhance system-level understanding and inform the development of potential interventions.

### Settings

A two-day GMB workshop was held in Phnom Penh from December 5th to 9th, 2021, in collaboration with the Khmer HIV/AIDS NGO Alliance (KHANA). We invited 28 stakeholders from various backgrounds (excluding the principal investigator and moderator) to participate in the GMB exercise, with 27 attending. KHANA identified and purposively sampled these stakeholders based on their personal and institutional experience with FEWs. Details of the stakeholder participants are provided in Supplementary Table S1.

The workshop was conducted in a hybrid format—via Zoom (38) and in person—due to difficulties reported by FEWs in attending via videoconferencing. The number of FEWs invited was reduced due to logistical constraints and COVID-19 restrictions on in-person gatherings. FEWs gathered at KHANA’s headquarters with KHANA facilitators for their participation in the GMB session.

### GMB exercise outline

Each day of the exercise featured a critical interactive activity. After presenting the agenda to stakeholders in the main room, participants were divided into three breakout rooms, each with a diverse mix of individuals. To ensure consistency across all groups, facilitators used a standardized script for each activity, adapted from Scriptopedia for the GMB approach. The facilitators were trained before the GMB exercise to maintain uniformity across breakout rooms. They encouraged active participation, ensuring that everyone had the opportunity to share their views. Participants elaborated on the factors they raised, while others were invited to provide additional input. The introduction and main discussions were conducted in English, with Khmer translation in the main room. During breakout sessions, discussions were held in Khmer, and facilitators translated the findings into English.

#### GMB exercise 1– variable elicitation

Day 1 involved a variable elicitation exercise to identify and discuss factors affecting the mental health of FEWs during the COVID-19 pandemic. The guiding question for stakeholders was: “*What factors, directly or indirectly, impact the mental health of FEWs due to the COVID-19 pandemic?*” Each group had 90 min for discussion. Facilitators sought clarifications to ensure a shared understanding and agreement among stakeholders before recording variables on a digital whiteboard. After the discussions, facilitators had 5 min to present and summarize their variables on the main room’s digital whiteboard. Variables elicited on Day 1 are depicted in Supplementary Figures S1a–c.

If any uncertainties remained, the modeler clarified the relationships between variables and mental health with stakeholders. By the end of Day 1, the modeler used notes from the variable elicitation exercise to construct a preliminary conceptual model using the bespoke software VenSim (39) based on their understanding of the discussions from Day 1.

#### GMB exercise 2– policy elicitation

On Day 2, a policy elicitation exercise was conducted to identify existing policies addressing the impact of the COVID-19 pandemic on FEWs’ mental health and to discuss potential interventions. The guiding questions for stakeholders were: “*What interventions have been in place that improved the mental health of FEWs since the COVID-19 pandemic began? What additional interventions could enhance FEWs’ mental health?*” Each group had 1 h for discussion, with facilitators recording the outcomes on a digital whiteboard. After the discussions, facilitators summarized the findings in a five-minute presentation. Policies identified on Day 2 are depicted in Supplementary Figures S2a–c.

The preliminary conceptual model was then presented to stakeholders to verify its accuracy and ensure it reflected their inputs. The modeler reviewed each causal loop with live translation provided by facilitators. Stakeholders’ feedback during the review led to significant modifications to the model. The CLD was then refined and consolidated through iterative analyses using notes from the GMB sessions, aligning the variables and pathways with the underlying causal logic expressed by participants.

### Ethics consideration

Participants received an invitation letter detailing the study’s objectives and procedures. To protect their identities, personal information, such as ID numbers, addresses, and phone numbers, was not collected. Comprehensive briefings and written documentation explained the study’s purposes, procedures, and potential risks. Informed consent was obtained through a form reviewed and signed by each participant, confirming their understanding and voluntary agreement to participate. Participants were informed that discussions in the main room would be recorded solely for refining the CLDs and were given the option to turn off their video cameras. Proxy names (e.g., P1, P2) were used to prevent linking identifiable information to sensitive experiences shared. Discussions focused on general factors and systemic aspects of mental health rather than personal anecdotes. The data collection team ensured confidentiality by not recording discussions within the breakout rooms. Participants were reimbursed for their travel costs and received a token of appreciation of USD 10 for participating in the GMB exercise.

## Results

### Participant characteristics

The workshop included 27 stakeholders: 17 (62.9%) were female, 17 (62.9%) were aged 21–40, 17 (62.9%) were from local NGOs, six (22.2%) were from FEW communities, three (11.1%) were from national institutions, and one (3.7%) was from an international NGO.

### Causal map of the impact of the COVID-19 pandemic on FEWs’ mental health

Based on the outcomes of the GMB exercise, we identified 13 feedback loops, including 11 reinforcing and two balancing loops. These feedback loops were categorized into three main themes: (1) COVID-19 and the fear of contracting COVID-19, (2) anxiety related to financial and economic situations, employment, and food security, and (3) lack of family support. Variables have been numbered for ease of reference and to facilitate tracing loops within the CLD.

#### COVID-19 pandemic and fear of contracting COVID-19

**Figure 1:**
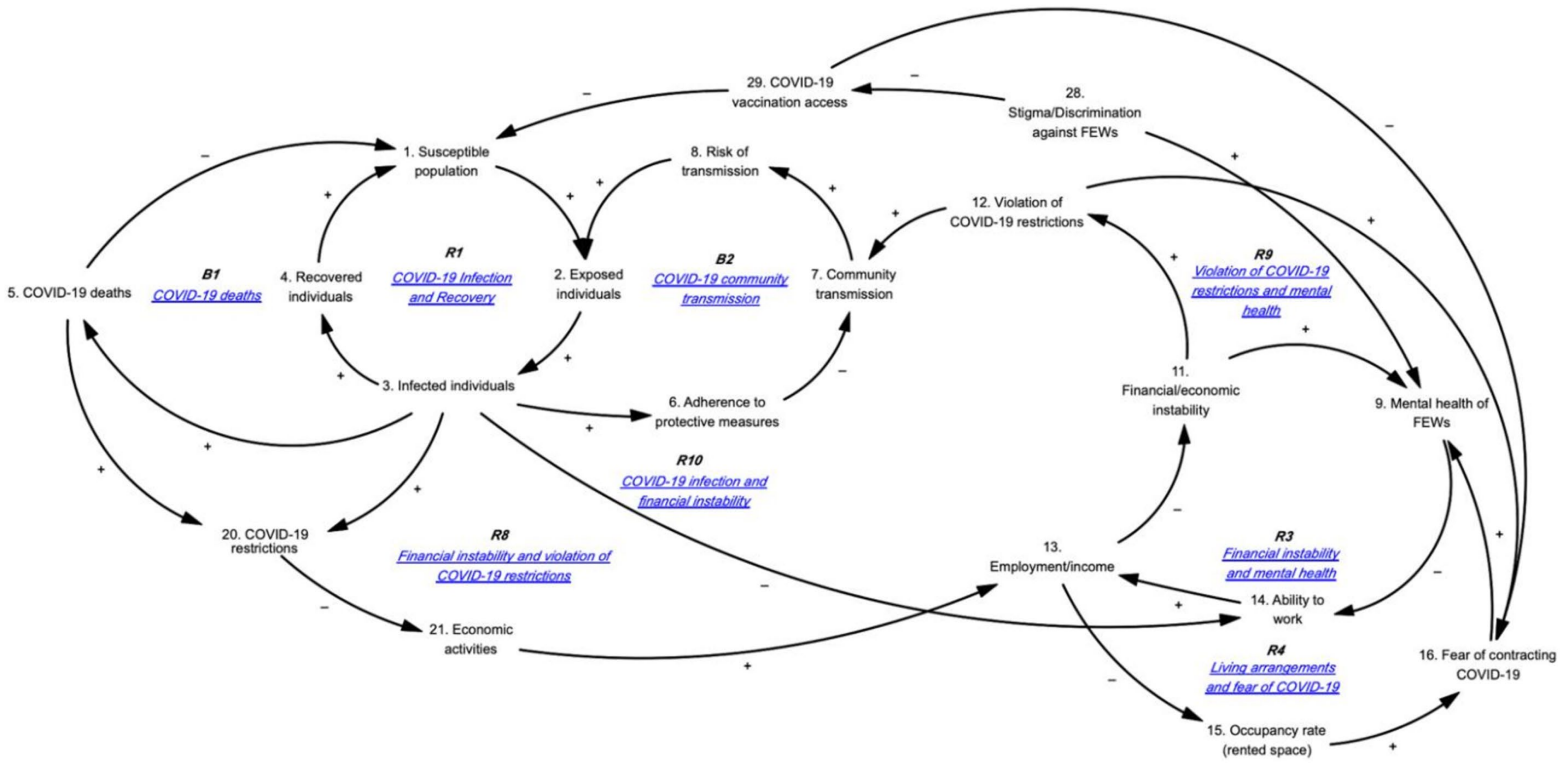
Impact of the COVID-19 pandemic and fear of contracting COVID-19 on mental health.

Figure 1 illustrates the feedback loops related to the impact of the COVID-19 pandemic and the fear of contracting COVID-19 on mental health. Table 2 provides the corresponding causal pathways. Reinforcing feedback loop R1 depicts the COVID-19 infection and recovery foundation. As the susceptible population grows, the number of exposed individuals increases, leading to more infections and, subsequently, more recoveries. Recovered individuals remain susceptible to reinfection, which enlarges the susceptible population. Balancing feedback loop B1 shows that as the susceptible population and the number of exposed and infected individuals rise, COVID-19-related deaths will increase, reducing the susceptible population. Additionally, as the number of exposed individuals rises, more people will adopt protective measures, decreasing community transmission and the subsequent risk of infection. Loop R1 is counteracted when a decrease in exposed individuals reduces transmission risk, forming balancing feedback loop B2.

**Table 2:** Loop pathways of Figure 1.

Loop reference	Loop name	Loop pathway
R1	COVID-19 infection and recovery	1 ➔ 2 ➔ 3 ➔ 4 ➔ 1
R3	Financial instability and mental health	9 ➔ 14 ➔ 13 ➔ 11 ➔ 9
R4	Living arrangements and fear of COVID-19	13 ➔ 15 ➔ 16 ➔ 9 ➔ 14 ➔ 13
R8	Financial instability and violation of COVID-19 restrictions	3 ➔ 20 ➔ 21 ➔ 13 ➔ 11 ➔ 12 ➔ 7 ➔ 8 ➔ 2 ➔ 3
R9	Violation of COVID-19 restrictions and mental health	12 ➔ 16 ➔ 9 ➔ 14 ➔ 13 ➔ 11 ➔ 12
R10	COVID-19 infection and financial instability	3 ➔ 14 ➔ 13 ➔ 11 ➔ 12 ➔ 7 ➔ 8 ➔ 2 ➔ 3
B1	COVID-19 deaths	1 ➔ 2 ➔ 3 ➔ 5 ➔ 1
B2	COVID-19 community transmission	2 ➔ 3 ➔ 6 ➔ 7 ➔ 8 ➔ 2

Reinforcing feedback loop R3 indicates that as mental health stressors increase, FEWs’ ability to work diminishes, leading to reduced income and heightened financial instability, which in turn exacerbates mental health stressors. Reinforcing feedback loop R4 shows that decreased income results in higher occupancy rates within rented spaces, heightening fears of contracting COVID-19 and increasing mental health stressors. This, in turn, reduces their ability to work, further decreasing their income. FEWs, often urban migrants who leave their families in rural areas for work, share their rented spaces with more people when facing reduced income or unemployment, increasing their risk of COVID-19 infection. The lack of social protection and reduced access to healthcare services heightens their fear of contracting COVID-19, adding to their stress. Additionally, stigma and discrimination against FEWs impede their access to COVID-19 vaccinations, further increasing their susceptibility to infection.

Reinforcing feedback loop R8 illustrates that as the number of infected individuals rises, COVID-19 restrictions tighten, reducing economic activities and subsequently affecting FEWs’ employment and income. This situation exacerbates FEWs’ financial instability and leads to increased violations of COVID-19 restrictions to earn money, heightening community transmission and the risk of infection. Reinforcing feedback loop R9 demonstrates how increased violations of COVID-19 restrictions by FEWs escalate their fears of contracting COVID-19, which heightens mental health stressors and reduces their ability to work. This decreased ability to work further diminishes income and financial stability, prompting more violations of COVID-19 restrictions. Participants reported that FEWs felt unsafe working without vaccination, leading to decreased revenue and increased stress.

Reinforcing feedback loop R10 reveals that as the number of infected individuals grows, FEWs’ ability to work declines due to stricter COVID-19 restrictions, closure of entertainment venues, and fewer clients. The decrease in income heightens financial instability, leading to increased violations of COVID-19 restrictions, further amplifying community transmission and the number of exposed and infected individuals.

#### Anxiety toward financial/economic situation, employment, and food security

**Figure 2:**
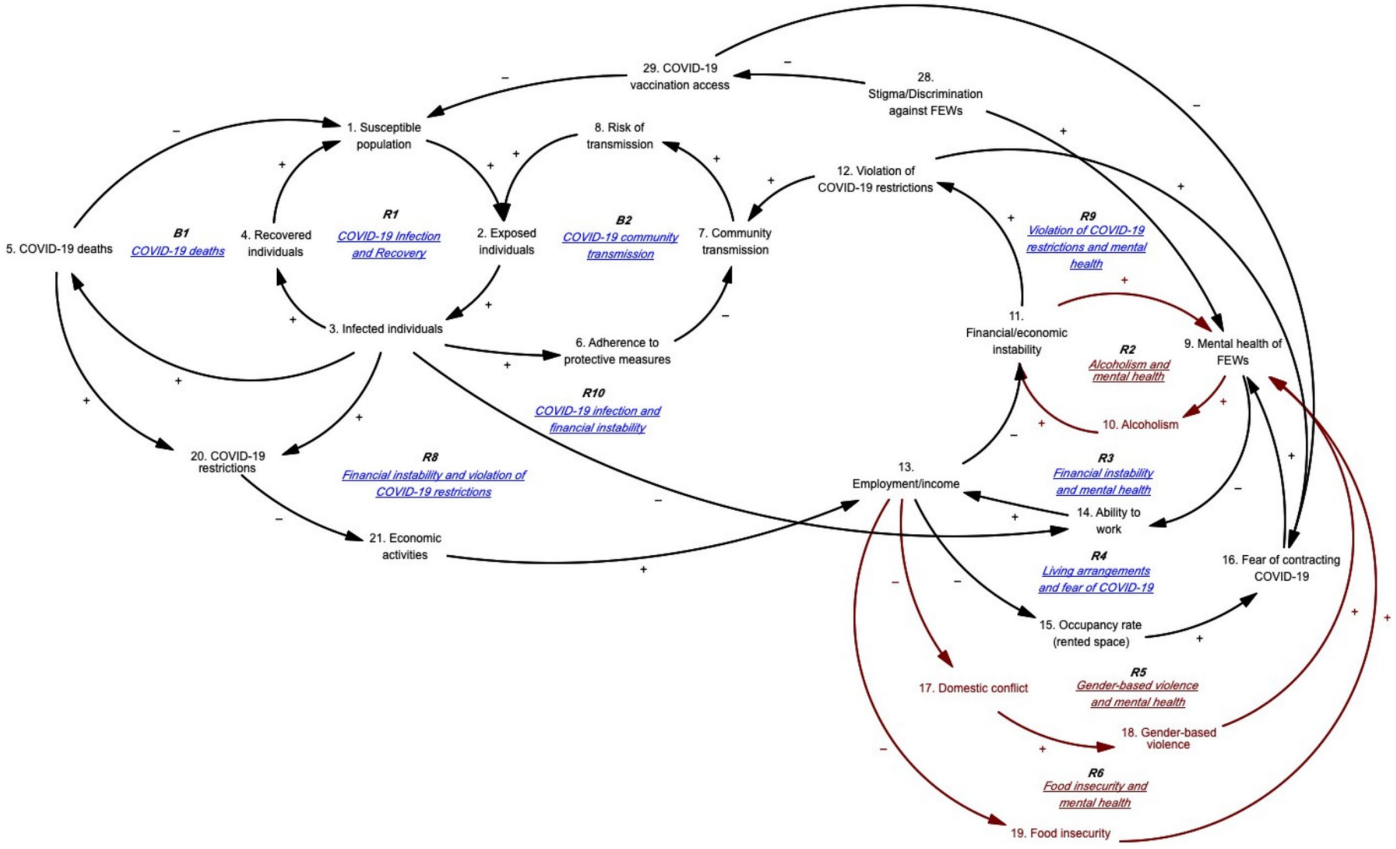
Anxiety toward financial/economic situation, employment, and food security.

Figure 2 illustrates the feedback loops depicting how anxiety related to financial situations, employment/income, and food security—exacerbated by the COVID-19 pandemic—affects the mental health of FEWs. Table 3 presents the corresponding causal pathways. Reinforcing feedback loop R2 highlights that as mental health stressors increase for FEWs, alcohol consumption also rises, which leads to higher expenditures and greater economic instability, further intensifying mental health stressors. FEWs reported turning to alcohol in response to mental health challenges, creating a vicious cycle of financial instability and worsening mental health.

**Table 3:** Loop pathways for Figure 2.

Loop reference	Loop name	Loop pathway
R2	Alcoholism and mental health	9 ➔ 10 ➔ 11 ➔ 9
R5	Gender-based violence and mental health	13 ➔ 17 ➔ 18 ➔ 9 ➔ 14 ➔ 13
R6	Food insecurity and mental health	13 ➔ 19 ➔ 9 ➔ 14 ➔ 13

Reinforcing feedback loop R5 hypothesizes that decreased income leads to increased domestic conflict and GBV. FEWs reported that reduced income exacerbates domestic tensions, resulting in higher rates of GBV, including physical, psychological, and sexual violence. As the primary income earners for their families, FEWs experience intensified mental health stressors due to these conflicts, which, in turn, decrease their ability to work and earn income. Many FEWs endure GBV both at home and from clients, driven by their precarious economic situations and the desire to avoid further conflicts with their families.

Stakeholders noted that employment and income instability significantly influence FEWs’ mental health, particularly during the pandemic. Some clients demanded rapid antigen tests without covering the cost, which added to FEWs’ financial strain and distress. With entertainment venues essentially closed, FEWs often violated COVID-19 restrictions to earn income despite the risk of contracting the virus. This led to harassment and arrests by law enforcement, further contributing to mental distress. The GMB exercise revealed that financial instability has a profound direct and indirect impact on FEWs’ mental health, with severe effects on their primary source of income and limited opportunities for alternative employment due to their backgrounds and lack of vocational training.

Similarly, reinforcing feedback loop R6 demonstrates that decreased income contributes to increased food insecurity, which heightens mental health stressors and reduces work capacity and revenue. The COVID-19 pandemic severely affected food security and access in Cambodia, with market closures in Phnom Penh in April 2021 and transportation challenges exacerbating food shortages (40). Stakeholders reported that food insecurity became a critical issue for FEWs during the period.

#### Lack of support from and for family

**Figure 3:**
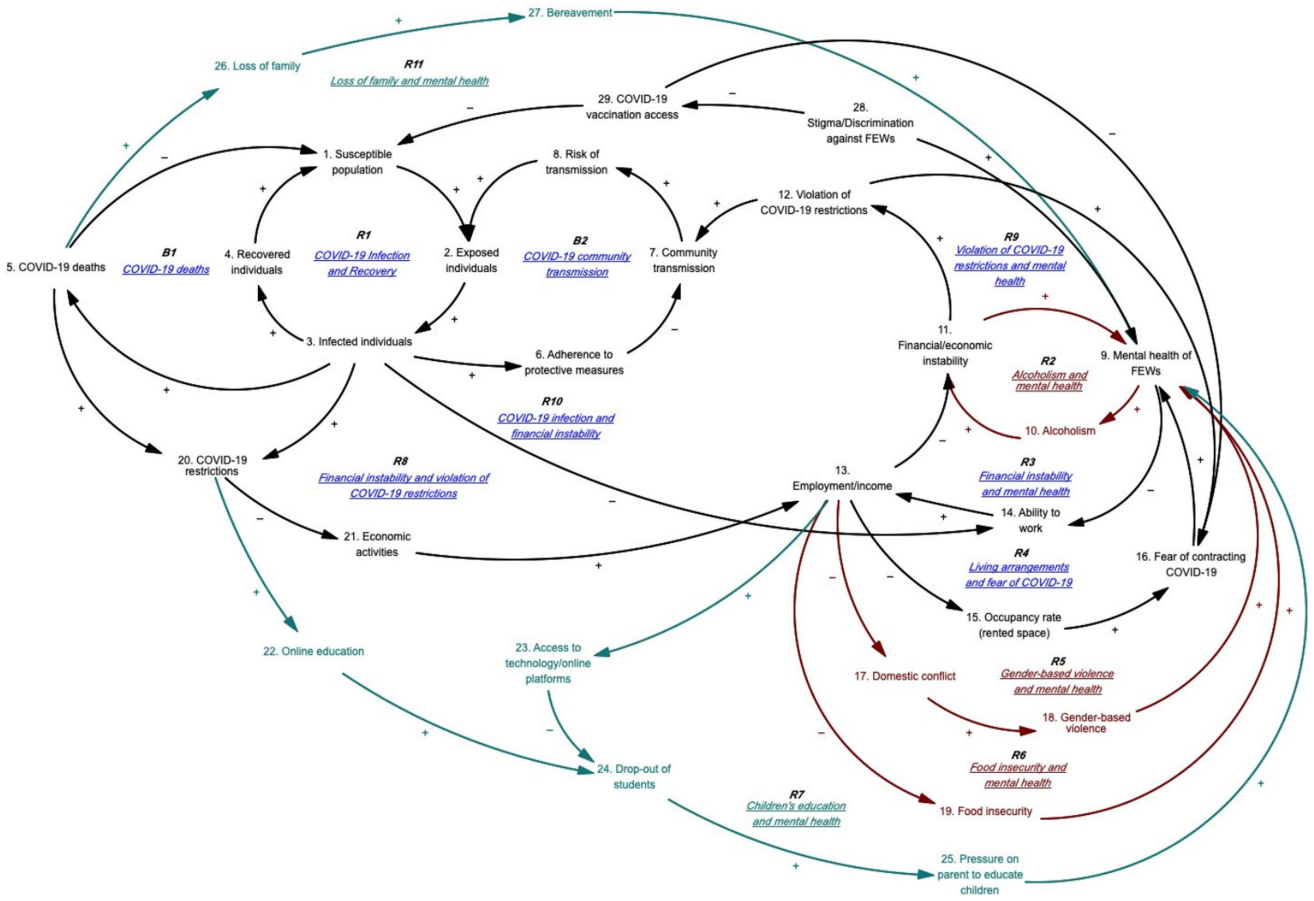
Lack of family support.

Figure 3 illustrates the feedback loops showing how family-related issues contribute to FEWs’ stressors. Table 4 details the corresponding pathways. Reinforcing feedback loop R7 indicates that as FEWs’ income decreases, their access to technology and online platforms diminishes. This leads to higher dropout rates among their children and an increased need for home-schooling, which intensifies FEWs’ mental health stressors. The time spent educating their children at home reduces their ability to work and earn income. During the COVID-19 pandemic, schools in Cambodia were closed for nearly two-thirds of the school year (250 days) in 2020 and 2021, resulting in significant learning loss (41). With the shift to online learning, FEWs faced difficulties providing their children with the necessary technology or access to online platforms due to financial constraints, leading to increased school dropout rates. Additionally, the responsibility for home-schooling often falls more heavily on women than men (42), exacerbating the stress for FEWs as they navigate the dual pressures of supporting their children’s education and maintaining their work and income.

**Table 4:** Loop pathways for Figure 3.

Loop reference	Loop name	Loop pathway
R7	Children’s education and mental health	13 ➔ 23 ➔ 24 ➔ 25 ➔ 9 ➔ 14 ➔ 13
R11	Loss of family and mental health	5 ➔ 26 ➔ 27 ➔ 9 ➔ 10 ➔ 11 ➔ 12 ➔ 7 ➔ 8 ➔ 2 ➔ 3 ➔ 5

Reinforcing feedback loop R11 demonstrates that as COVID-19-related deaths rise, FEWs experience an increase in the loss of family members, leading to heightened bereavement and mental health stressors. Domestic travel restrictions exacerbated this situation, leaving FEWs feeling unsupported by their families. Those who lost family members to COVID-19 were often unable to return to see them in their final moments. This inability to grieve appropriately and say a final farewell intensified feelings of social isolation and worsened their mental health.

## Discussion

Our findings underscore how pre-existing issues faced by FEWs were exacerbated by the pandemic and reveal factors that may not have been previously identified in studies on the mental health of FEWs and FSWs. This study offers a comprehensive understanding of the diverse and interconnected factors impacting FEWs’ mental health. Key stressors identified by stakeholders include financial instability, which was worsened by the pandemic, and anxiety related to COVID-19. Concerns about income and employment were linked to various other stressors that aggravated mental health issues. These findings align with past research highlighting stressors such as exposure to GBV, concerns about mental and physical health, and threats of arrest (14). They also reaffirm the close relationship between FEWs’ mental health and their physical and occupational health (7), indicating that policies or programs addressing these areas are likely to impact all three aspects.

LMICs, including Cambodia, have faced significant challenges in developing mental healthcare systems due to factors such as insufficient resources and funding, lack of political will, and low prioritization of mental health compared to other public health issues (43). In Cambodia, the destruction of health infrastructure and services during the Khmer Rouge period has severely hindered the rebuilding of mental health services (44). The absence of mental health legislation (44) and poor policy implementation further delayed the development of the mental health system. Participants in our GMB exercise highlighted issues such as the lack of awareness and significant stigma associated with mental health diagnoses in Cambodia (43), which are consistent with previous research.

Stigma against FEWs and mental disorders exacerbates the difficulties FEWs encounter in accessing mental health services. Participants also pointed out issues related to service availability and quality, reflecting previous findings of gaps in mental healthcare (43). Marginalized populations like FEWs face even more significant challenges in accessing the already limited mental health services available to the general population. The pandemic has further reduced the availability of these services by redirecting healthcare resources, making it even less likely that vulnerable communities will receive the mental health support they need. The high burden of mental health issues among FEWs highlights the urgent need for prevention and treatment services, as well as improvements in work environments to enhance mental health outcomes in the long term. This study provides a foundation for policymakers, mental healthcare providers, and organizations to explore mental healthcare provision and related factors to improve the overall mental health of FEWs.

### Policy implications

We identified four main concerns stakeholders felt were most affected by the COVID-19 pandemic: employment/income, GBV, food insecurity, and access to mental health services. Consequently, four critical interventions were proposed to address these issues and improve the mental health of FEWs.

Employment and income were identified as critical leverage points impacting the mental health of FEWs. Stakeholders suggested that financial aid could significantly alleviate stressors related to instability in employment and income during emergencies. However, they also acknowledged that financial aid is a short-term solution and may be constrained by limited resources. To address this, they recommended that the government and development partners provide vocational training for FEWs, enabling them to pursue alternative employment opportunities. Such training could help FEWs transition out of the entertainment and informal sex work industries, potentially breaking intergenerational cycles of poverty and improving mental health and overall well-being.

Stakeholders highlighted a significant gap in mental health services, which was exacerbated by the COVID-19 pandemic. Even before the pandemic, FEWs faced considerable barriers to accessing care due to discrimination and stigmatization, impeding their ability to obtain necessary mental health support (45). Addressing these gaps requires fostering multi-sectoral collaboration between government and NGOs to drive meaningful improvements in mental health care. Government resources should be strategically allocated to expand general and mental health services, and community-based organizations should be utilized for outreach to identify at-risk FEWs and connect them with available support services. However, implementing these interventions involves several challenges, including limited funding, a shortage of trained mental health professionals, and community resistance due to stigma. Overcoming these barriers will necessitate substantial resources, including financial investment, training programs for healthcare providers, and public awareness campaigns to combat stigma. Additionally, integrating health screenings to assess and address occupational risk factors into existing health services will require careful coordination and resource allocation.

To address these challenges, a phased approach could be adopted, starting with pilot programs to test the feasibility of proposed interventions. These pilots should be accompanied by robust evaluation frameworks to assess their effectiveness and make necessary adjustments. Collaborating with international organizations and leveraging existing infrastructure will be crucial for mobilizing resources and expertise. By addressing these factors, it will be possible to implement the recommended interventions effectively and achieve meaningful improvements in mental health care for FEWs.

Lastly, GBV remains a pervasive issue among FEWs and requires urgent attention. The pandemic has intensified both global and Cambodian incidences of GBV (46). The Cambodian Centre for Human Rights reports that economic pressures heighten the risk of GBV, leading some FEWs into exploitative or abusive situations driven by financial necessity (47). This issue, identified in the GMB as a significant driver of mental health problems among FEWs, is further exacerbated by stigma and harassment from local authorities due to their job nature. Participants have emphasized the need for prompt responses from authorities when GBV incidents are reported to ensure the protection of FEWs. It is crucial to investigate existing processes within law enforcement agencies to identify challenges officers face in handling GBV reports. Once these barriers are identified, tailored interventions should be developed to enhance response times to GBV cases. Additionally, comprehensive training programs are needed to equip law enforcement officers with skills to engage with women in a gender-sensitive and trauma-informed manner.

To further substantiate our findings, future cost–benefit analyses should be conducted to quantitatively assess the financial implications of the strategies identified through the GMB. Collecting data on the costs of various public health interventions or policies and simulating their long-term health and economic impacts using the GMB framework will provide robust, quantitative evidence. This evidence will support more informed decision-making by clarifying the trade-offs between intervention strategies, highlighting potential barriers, and identifying necessary resources for effective implementation.

### Strengths and limitations

The GMB exercise provided a platform for participants from diverse backgrounds, experiences, and age groups to openly discuss their perceptions of mental health issues—a topic that is often socially stigmatized within this vulnerable population in Cambodia. The findings are valuable for generating new hypotheses and pinpointing areas for intervention. The causal map illustrating the impact of the COVID-19 pandemic on FEWs’ mental health offers stakeholders, policymakers, and organizations developing programs for FEWs a deeper understanding of the contextual factors affecting their mental well-being. This map also highlights leverage points for strengthening and improving mental health services for FEWs. Additionally, it serves as a visual tool that demonstrates the complex interrelationships among the various factors influencing FEWs’ mental health.

This study has several limitations. Firstly, the FEWs involved may not fully represent the broader FEW populations in Cambodia, as they were primarily individuals already receiving aid through KHANA’s community-based projects. Due to COVID-19 restrictions, we could include only a limited number of participants. Future research should aim to include a more extensive and diverse sample from various regions in Cambodia, including those not receiving aid, to improve representation and reduce bias. Secondly, despite efforts to de-identify participants, there may have been pressure to disclose sensitive experiences in large groups, potentially leading to the under-reporting of certain variables. Additionally, the hybrid nature of the exercise, coupled with language barriers and time constraints, may have resulted in the loss of specific perspectives during translation, particularly in model conceptualization.

Lastly, while GMB enhances stakeholder engagement and understanding, it has notable limitations. The process is time-intensive, requiring significant effort to organize, facilitate, and synthesize group input, which can delay decision-making in urgent situations. Effective facilitation is crucial; without it, group dynamics may negatively impact model outcomes. However, facilitator training can help mitigate this issue. The collaborative nature of GMB can sometimes lead to groupthink, where dominant voices skew the model. Trained facilitators work to ensure balanced participation. Aligning diverse stakeholders with varying priorities can also be challenging, but focusing on shared outcomes and revising the model through iterations helps achieve consensus.

Translation and interpretation challenges were carefully managed while conceptualizing the CLDs to ensure the accuracy and relevance of the data. Experienced translators and interpreters, well-versed in both the technical language of the study and the local context, were employed to minimize misinterpretations and capture the nuances of the discussions accurately. Facilitators were also trained to address potential language barriers during sessions, and multiple rounds of review were conducted to verify the accuracy of the translated data. Possible impacts of translation and interpretation issues include the risk of miscommunication, which could lead to inaccurate representations of stakeholder inputs and introduce biases into the CLDs.

To mitigate these risks, we implemented several measures: regular feedback loops between translators and facilitators, iterative verification of translated content with participants, and cross-checking key concepts for consistency across languages. These steps aimed to maintain data integrity and ensure that the final CLDs accurately reflected participants’ inputs. In the analyses, we addressed ambiguities by incorporating iterative validation processes and seeking expert input. Stakeholders and experts reviewed and provided feedback on the CLDs, helping to clarify and refine the causal relationships. Additionally, sensitivity analyses were conducted to explore how changes in causal assumptions might affect overall model outcomes. These measures were designed to ensure that the CLDs accurately represented the complex dynamics of the system while acknowledging and mitigating potential ambiguities.

## Conclusion

This study identified several key factors affecting the mental health of FEWs, an HIV key population in Cambodia, during the COVID-19 pandemic. These factors include bereavement, financial instability, fear of contracting COVID-19, child homeschooling responsibilities, and food insecurity. The study highlights potential interventions to address these challenges, such as vocational training, mental health awareness programs, and efforts to combat GBV within this vulnerable population. The study underscores the importance of recognizing the complex interplay of these factors and their impact on FEWs’ mental health, providing valuable insights for future interventions and policies. It emphasizes the global importance of prioritizing mental health, given its connections to physical health, and calls for improved access to mental health services for FEWs, including telehealth options, while acknowledging existing gaps in service coverage and quality. Additionally, this study suggests the need for formal evaluations of proposed interventions before their implementation. Future quantitative research is essential to validate and complement these findings, providing more detailed and generalizable data on the factors affecting FEWs’ mental health and helping to refine the proposed interventions.

## Data Availability

The raw data supporting the conclusions of this article will be made available by the authors, without undue reservation.
