# Supplementary material for: Assessing the impact of the COVID-19 pandemic on the mental health of female entertainment workers in Cambodia: a group model building approach
Source: Front Public Health. 2024 Nov 21;12:1336785. doi: 10.3389/fpubh.2024.1336785 (PMC11617363; doi:10.3389/fpubh.2024.1336785)
Supplement: Supplementary file 1 [file Data_Sheet_1.PDF]

Supporting information

Table S1. Details of organizations represented

**Name of organization**	**Type of organization**	**Number of stakeholders (*n*=27)**
Female entertainment worker	NA	6
Cambodia Women for Peace and Development (CWPD)	Local non-governmental organization	9
Joint United Nations Program on HIV/AIDS (UNAIDS)	International non-governmental organization	1
Khmer HIV/AIDS NGO Alliance (KHANA)	Local non-governmental organization	8
National AIDS Authority (NAA)	National institution – a multi-sectoral body of the Royal Government of Cambodia	1
National Center for HIV/AIDS, Dermatology and STD (NCHADS)	Operational unit of the Ministry of Health Cambodia	2

Supporting information

Table S2. Loop pathways and references

Loop Reference	Loop name	Loop pathway	Policies
R1	COVID-19 infection and recovery	1→2→3→4→1	
R2	Alcoholism and mental health	9→10→11→9	Mental health services
R3	Financial instability and mental health	9→14→13→11→9	Financial aid<br>Alternative employment/vocational training<br>Mental health services
R4	Living Arrangements and fear of COVID-19	13→15→16→9→14→13	Financial aid<br>Mental health services
R5	Gender-based violence and mental health	13→17→18→9→14→13	Quick response from authorities<br>Mental health services
R6	Food insecurity and mental health	13→19→9→14→13	Food aid<br>Mental health services
R7	Children's education and mental health	13→23→24→25→9→14 →13	Financial aid<br>Governmental support
R8	Financial instability and violation of COVID-19 Restrictions	3→20→21→13→11→12 →7→8→2→3	
R9	Violation of COVID-19 restrictions and mental health	12→16→9→14→13→11 →12	Alternative employment vocational training

Supporting information

R10	COVID-19 infection and financial instability	3→14→13→11→12→7→8→2→3	
R11	Loss of family and mental health	5→26→27→9→10→11→12→7→8→2→3→5	Mental health services (including telehealth)
B1	COVID-19-related deaths	1→2→3→5→1	
B2	COVID-19 community transmission	2→3→6→7→8→2	

Supporting information

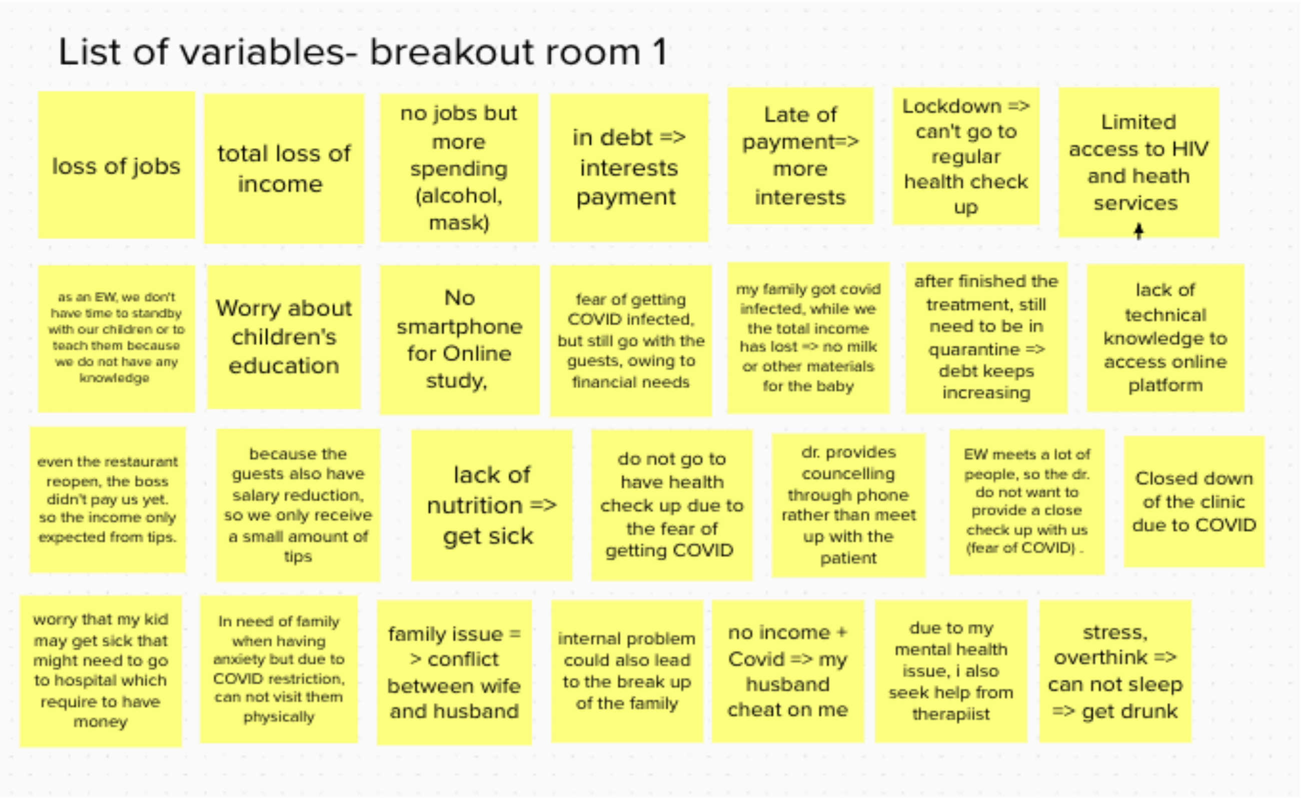

Figure S1a. Variables from breakout room 1

Supporting information

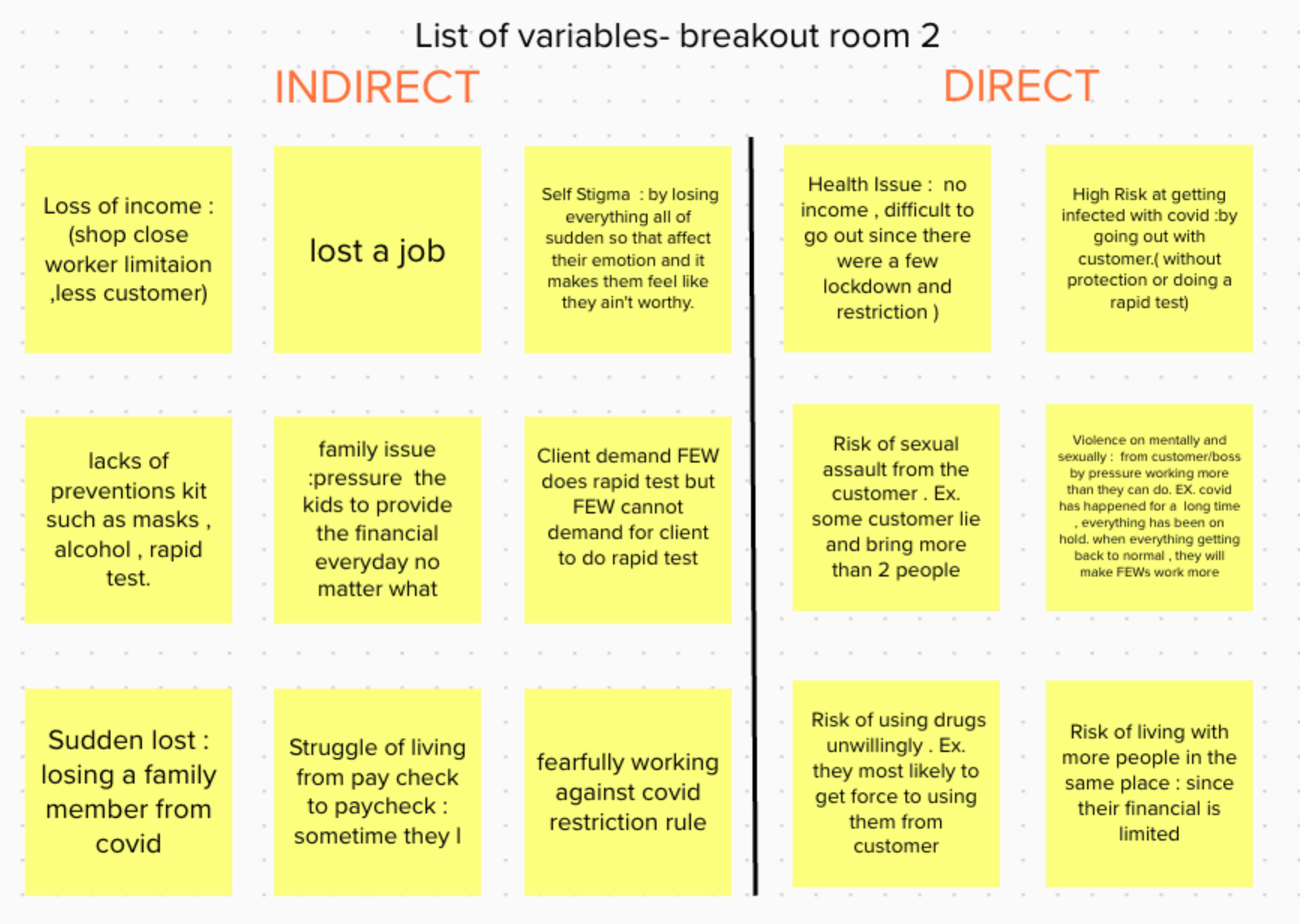

Figure S1b. Variables from breakout room 2

Supporting information

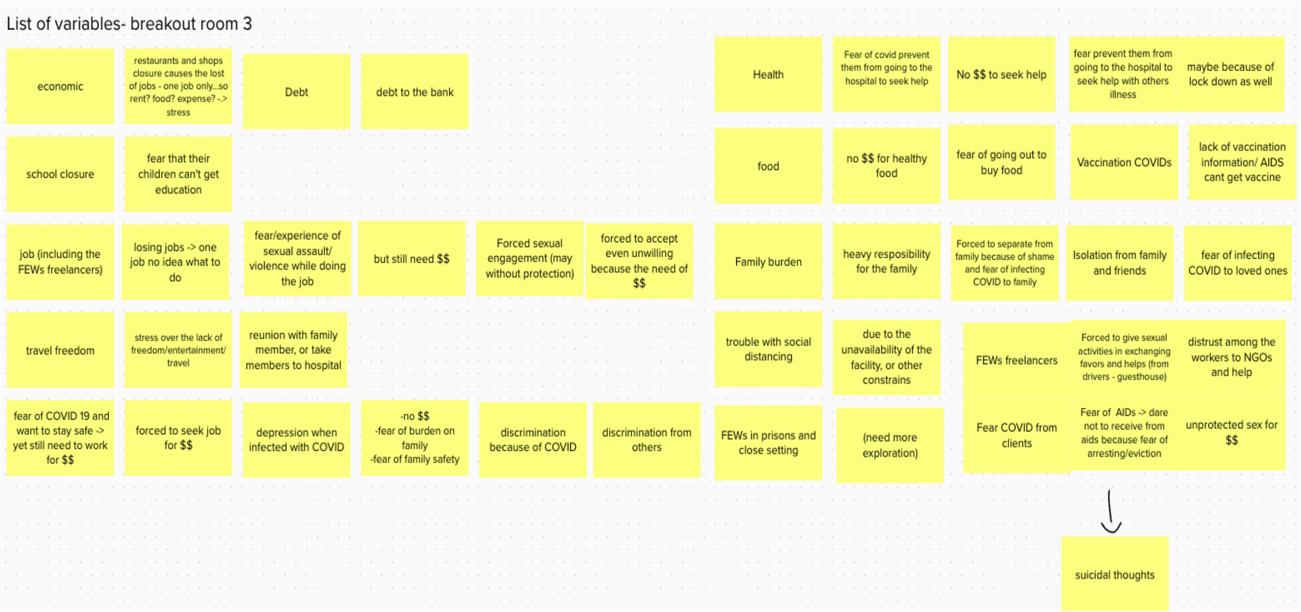

Figure S1c. Variables from breakout room 3

Supporting information

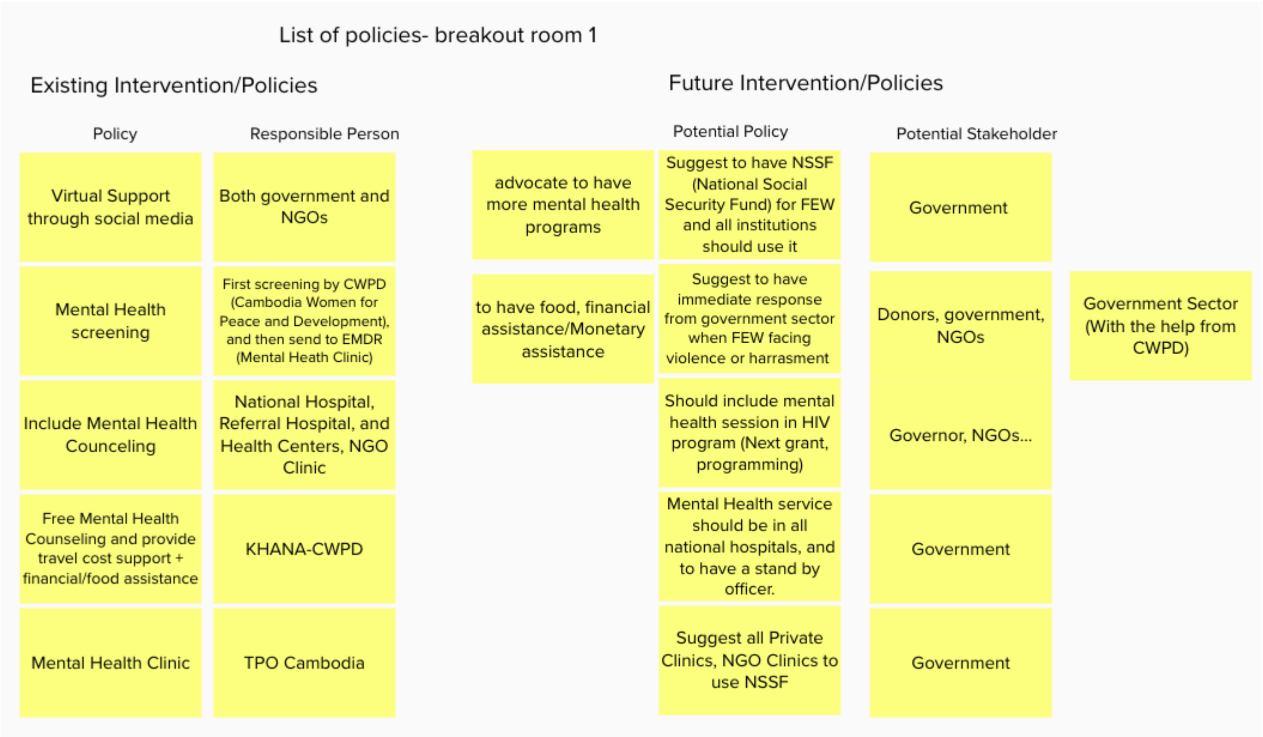

Figure S2a. Policies from breakout room 2

Supporting information

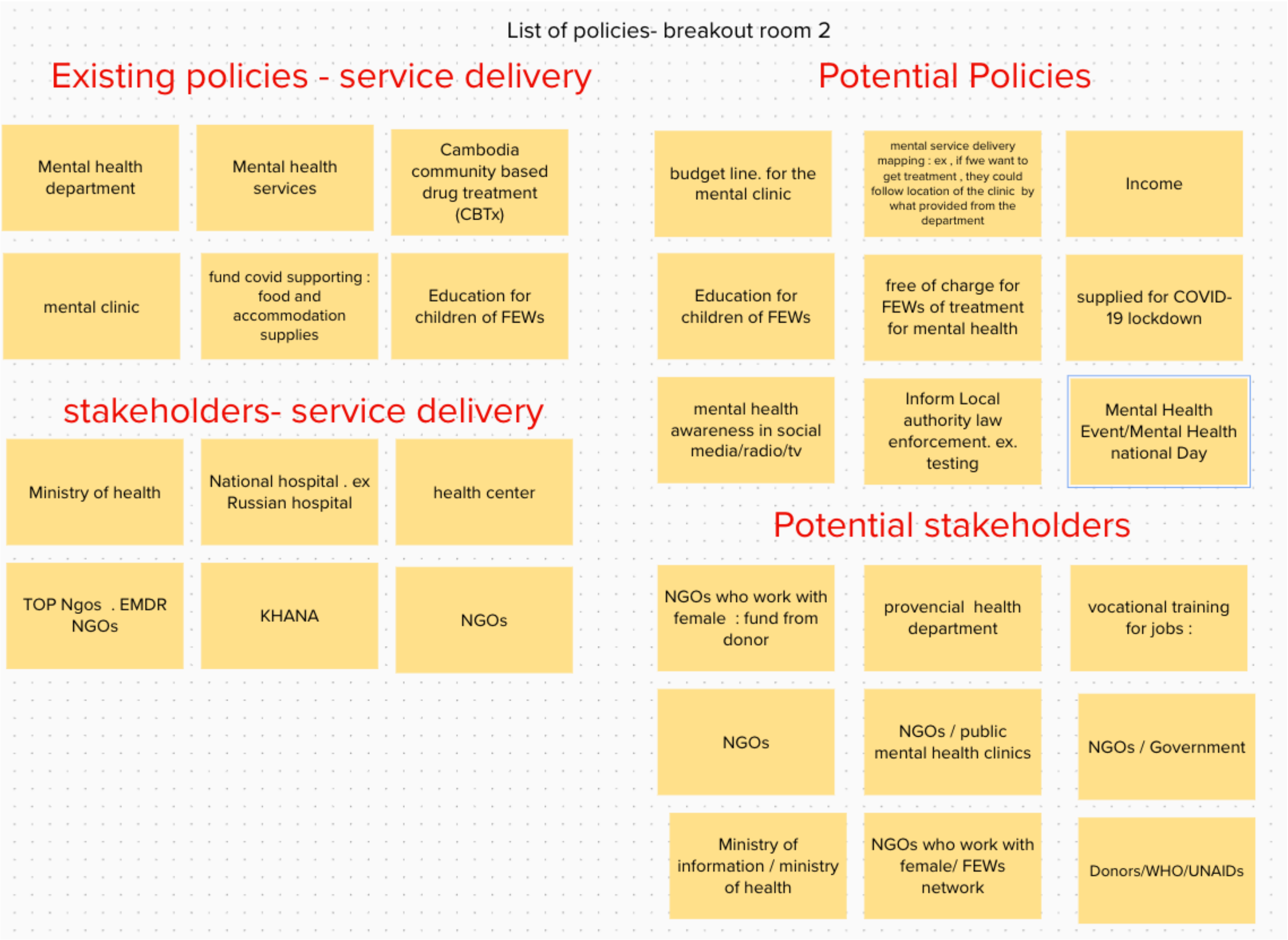

Figure S2b. Policies from breakout room 2

Supporting information

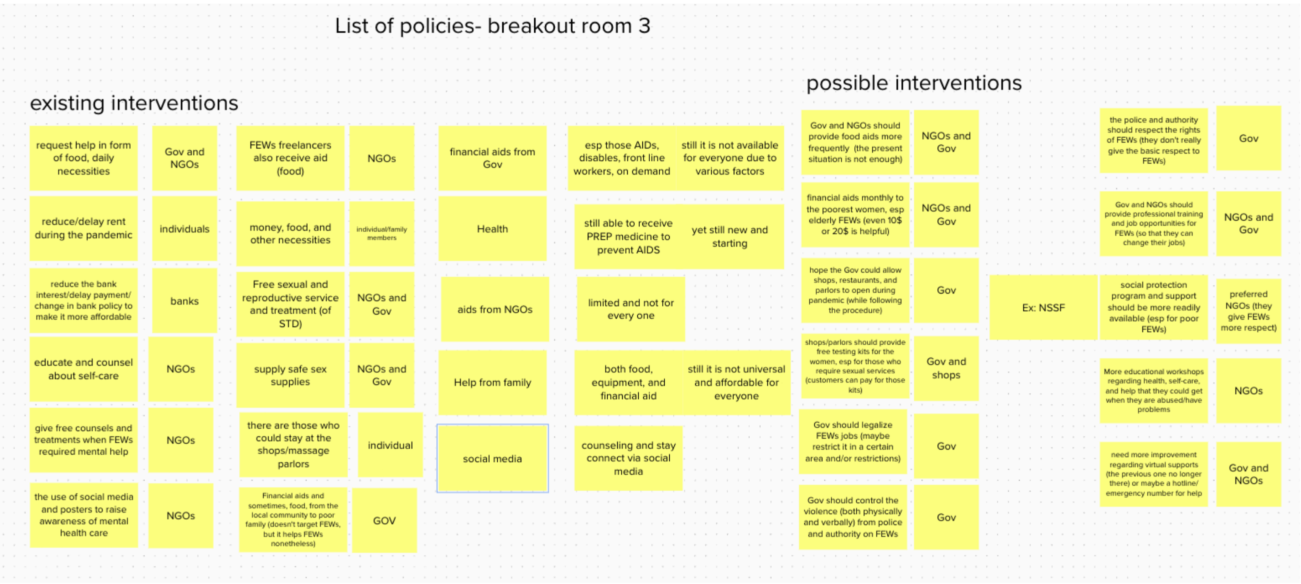

Figure S2c. Policies from breakout room 3
